# Integrated Analysis of miRNA and mRNA Endorses a Twenty miRNAs Signature for Colorectal Carcinoma

**DOI:** 10.3390/ijms20164067

**Published:** 2019-08-20

**Authors:** Andrea Angius, Paolo Uva, Giovanna Pira, Maria Rosaria Muroni, Giovanni Sotgiu, Laura Saderi, Elena Uleri, Maurizio Caocci, Gabriele Ibba, Maria Rosaria Cesaraccio, Caterina Serra, Ciriaco Carru, Alessandra Manca, Francesca Sanges, Alberto Porcu, Antonia Dolei, Antonio Mario Scanu, Paolo Cossu Rocca, Maria Rosaria De Miglio

**Affiliations:** 1 Istituto di Ricerca Genetica e Biomedica (IRGB), CNR, Cittadella Universitaria di Cagliari, 09042 Monserrato (CA), Italy; 2 CRS4, Science and Technology Park Polaris, Piscina Manna, 09050 Pula (CA), Italy; 3 Department of Biomedical Sciences, University of Sassari, Viale San Pietro 43-b, 07100 Sassari, Italy; 4 Department of Medical, Surgical and Experimental Sciences, University of Sassari, Viale San Pietro 8, 07100 Sassari, Italy; 5 Department of Prevention, Registro Tumori Provincia di Sassari, ASSL Sassari-ATS Sardegna, Via Rizzeddu 21, 07100 Sassari, Italy; 6 Department of Pathology, AOU Sassari, Via Matteotti 60, 07100 Sassari, Italy; 7 Department of Diagnostic Services, “Giovanni Paolo II” Hospital, ASSL Olbia-ATS Sardegna, Via Bazzoni-Sircana, 07026 Olbia, Italy

**Keywords:** Colorectal cancer, miRNA-mRNA interactions, MicroRNA expression profile, ACSL6, PRPS1, PRPS2, WNT signaling pathway, Hippo signaling pathway, Apelin signaling pathway, Ferroptosis

## Abstract

Colorectal cancer (CRC) ranks as the most frequent carcinoma worldwide. CRC patients show strong prognostic differences and responses to treatment, and 20% have incurable metastatic disease at diagnosis. We considered it essential to investigate mechanisms that control cellular regulatory networks, such as the miRNA–mRNA interaction, known to be involved in cancer pathogenesis. We conducted a human miRNome analysis by TaqMan low density array, comparing CRC to normal colon tissue (NCT, and experimentally identified gene targets of miRNAs deregulated, by anti-correlation analysis, with the CRC whole-transcriptome profile obtained from RNASeq experiments. We identified an integrated signature of 20 deregulated miRNAs in CRC. Enrichment analyses of the gene targets controlled by these miRNAs brought to light 25 genes, members of pathways known to lead to cell growth and death (*CCND1, NKD1, FZD3, MAD2L1,* etc.), such as cell metabolism (*ACSL6, PRPS1-2*). A screening of prognosis-mediated miRNAs underlined that the overexpression of miR-224 promotes CRC metastasis, and is associated with high stage and poor survival. These findings suggest that the biology and progression of CRC depend on deregulation of multiple miRNAs that cause a complex dysfunction of cellular molecular networks. Our results have further established miRNA–mRNA interactions and defined multiple pathways involved in CRC pathogenesis.

## 1. Introduction

Colorectal cancer is the most frequent cancer and the third most common cause of cancer-related mortality worldwide [1]. CRC is a well-studied malignancy for which extensive genetic abnormalities have been described, with well-defined risk factors, slow progression, and identifiable and curable preneoplastic lesions. Although the five-year survival rate is as high as 90% in patients with stage I disease, a dramatic reduction is seen to slightly larger than 10% in patients with stage IV disease [2]. Approximately 20% of patients with CRC already have metastases at diagnosis, and metastatic CRC is mainly an incurable disease [3]. CRC is considered a heterogeneous multifactorial disease showing drastically different prognoses and responses to treatment, indicating the relevance of understanding specific pathway abnormalities to improve diagnosis, prognosis and therapeutic strategies.

MicroRNAs (miRNAs), a family of endogenous small non-coding RNA, are involved in the regulation of multiple biological processes and metabolism [4,5]. The dysregulation of their expression is strongly related to development of cancer through the alteration of biological functions [6]. The recognition of miRNAs as regulators of gene expression has identified them as the most investigated epigenetic elements involved in cancer, so they could potentially be used as therapeutic targets, as well as diagnostic and prognostic biomarkers for malignancy. MiRNA expression level variations have been identified between normal and neoplastic colorectal tissues. Several lines of evidence from in vitro and nude mice models have shown that anti-cancer “miRNA mimics” inhibit cell proliferation, migration and induce apoptosis in CRC [7,8]. Several miRNAs are indicative of response to chemotherapy [9], and some have also shown prognostic potential in CRC [10]. Then, miRNAs might have a critical clinical significance in diagnosis, treatment and prediction of outcomes in CRC patients.

Our study performed an extensive analysis of miRNA expression profiles in CRC versus normal colon tissue to yield further insights to clarify the molecular mechanisms of oncogenesis in CRC. We experimentally identified, by whole-transcriptome profiling, gene targets for deregulated miRNAs to improve therapeutic strategies in CRC patients.

## 2. Results

### 2.1. Integrated Signature of miRNAs in CRC

**Figure 1:**
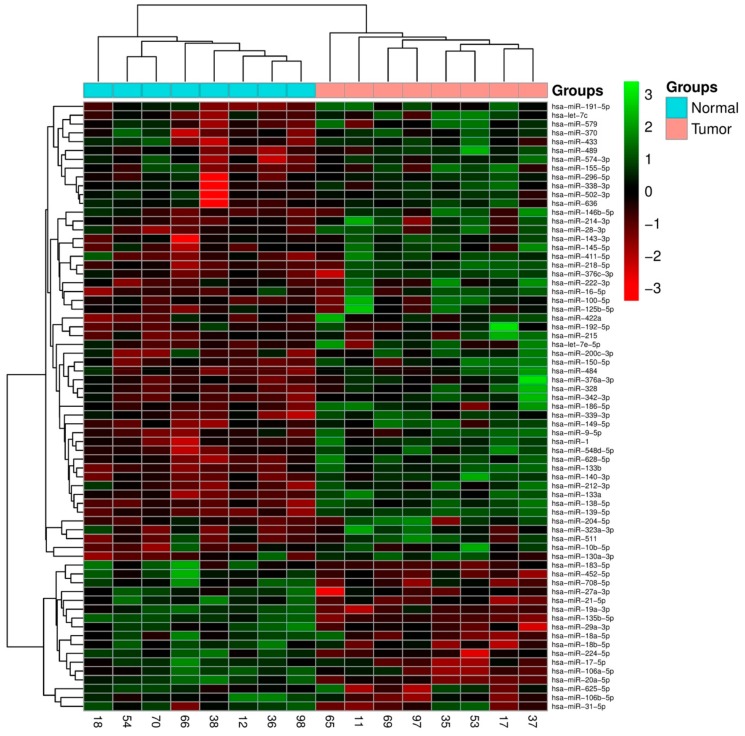
A 67-miRNA expression signature reveals changes between colorectal cancer (CRC) and NCT. Unsupervised hierarchical clustering analysis of CRC (blue) and NCT (pink) was performed using 67 differentially expressed miRNAs. Dendrograms of clustering analysis for samples and miRNAs are displayed on the top and left, respectively, and depict similarities in the miRNA expression profiles among the samples. The relative up and down regulation of miRNAs is indicated by red and light green, respectively. hsa; *Homo sapiens*. Endogenous control assays are present (MammU6, RUN44, and RUN48).

The expression levels of 377 miRNAs were acquired in CRC and NCT using TaqMan^®^ Array Human MicroRNA technology (TLDA). The statistical analysis of microarray (SAM) identifies 67 miRNAs (*p* value < 0.05) differentially expressed between the two categories, which showed a well-defined separation between normal colon and tumor tissues (Figure 1).

Predicted and known targets of all deregulated miRNAs were compared with whole-transcriptome profiles of CRC and NCT, obtained by RNA-Seq (G.P.; P.U. Landscape of transcriptome variations uncovering known and novel driver events in colorectal carcinoma. Sci Rep, under review), to identify miRNAs significantly enriched in anti-expressed targets.

A set of 21 miRNAs was identified (*p* value < 0.05): 20 were downregulated and only miR-224-5p was found upregulated in CRC (Table S1: List of 21 deregulated miRNAs identified by array and gene targets anti-expressed identified by RNA-Seq).

**Figure 2:**
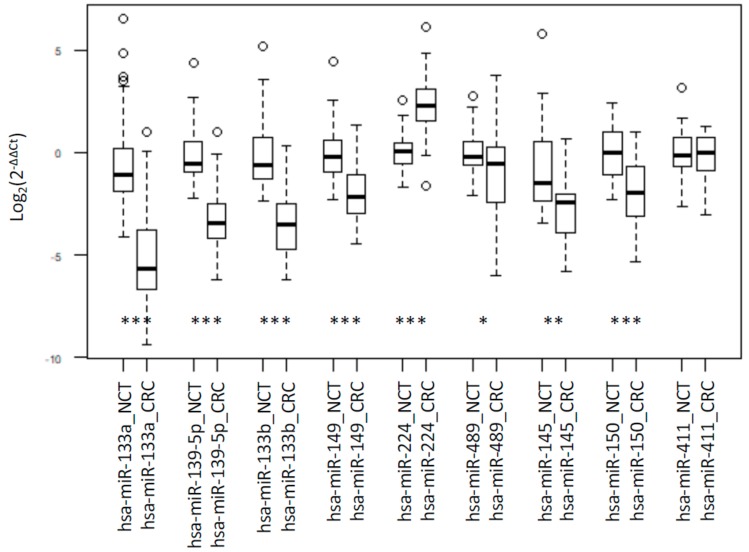
Box plot of RT-PCR results. RT-PCR quantification of miRNAs. The box border represents the interquartile range, the horizontal line in the box is the median, and circles represent outliers. Values are expressed as Log2(2^−ΔΔCt^). *, ** and *** represent significant differences between CRC and NCT at *p* value < 0.05, < 0.01 and < 0.001, respectively.

From this set, nine miRNAs showed a strong differential expression on microarray data (*p* value < 0.01) (miR-489, miR-139-5p, miR-133a, miR-133b, miR-411-5p, miR-145-5p, miR-150-5p, miR-149-5p, miR-224-5p) and were selected for experimental validation. RT-qPCR confirmed the differential expression data, except for miR-411-5p (Figure 2). 

A correlation analysis between the validated eight miRNAs showed significant positive correlation between all miRNAs downregulated in CRC, except for miR-489. Moreover, a strongly negative correlation was seen between miR-224 and 133a (*r* = 0.52, *p* < 0.001), miR-224 and miR-139-5p (*r* = 0.44, *p* < 0.05), miR-224 and miR-133b (*r* = 0.34, *p* < 0.05) (Table 1). The contemporary deregulation of these seven miRNAs in CRC might strongly influence the biology of this tumor.

### 2.2. Gene Targets of miRNAs and Functional Analysis

The comparison of differentially expressed (DE) miRNAs with whole-transcriptome profiles of CRC and NCT [11] allowed us to identify a set of 21 miRNAs enriched in anti-expressed targets (Table S1: List of 21 deregulated miRNAs identified by array and gene targets anti-expressed identified by RNA-Seq).

We then performed an enrichment analysis of anti-expressed targets to elucidate the biological function and molecular pathways possibly involved. The gene targets of miR-224-5p, the only one upregulated in CRC, affect three Gene Ontology (GO) biological processes: cellular monovalent inorganic cation homeostasis (*CHP2, MAFG, NEDD4L, SLC4A4*), regulation of bi-cellular tight junction assembly (*FZD5, NEDD4L*), and regulation of pH (*CHP2, MAFG, SLC4A4*). Meanwhile, targets of downregulated miRNAs were enriched in 37 KEGG pathways, 1428 GO Biological Processes and 206 GO Molecular Function (Table S2: The KEGG Pathways of downregulated miRNA gene targets in colorectal carcinoma; Table S3: The enriched Gene Ontology (GO) biological process categories of downregulated miRNA gene targets in colorectal carcinoma; Table S4: The enriched Gene Ontology (GO) molecular function categories of downregulated miRNA gene targets in colorectal carcinoma).

As a result, downregulated miRNAs affect important biological processes through the deregulation of their gene targets, such as epithelial development, protein export from the nucleus, and cell cycle process (see the full list in Table S3: The enriched Gene Ontology (GO) biological process categories of downregulated miRNA gene targets in colorectal carcinoma)). Deregulation of these basic biological processes may explain the molecular mechanisms of tumorigenesis and CRC biology.

**Figure 3:**
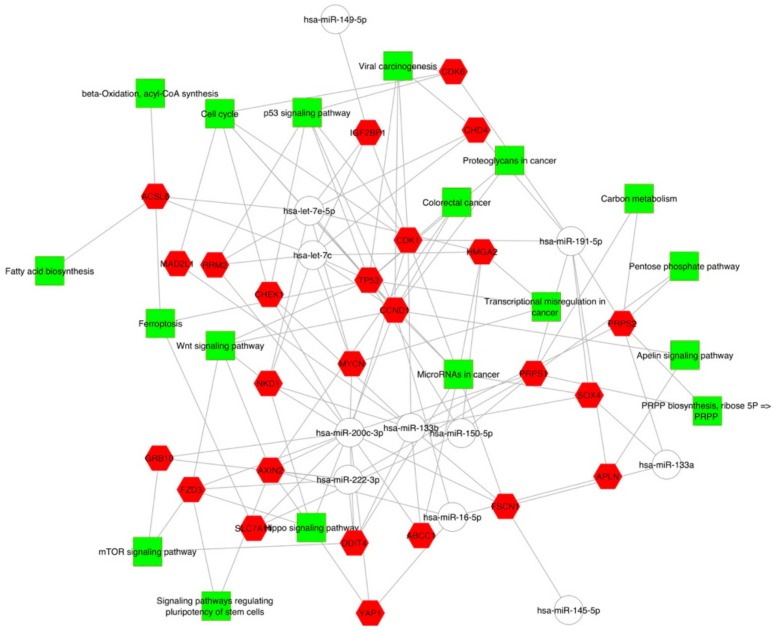
Colorectal tumor interactome network developed using Cytoscape. The gene targets were first submitted to Gene Ontology and KEGG pathway enrichment analysis using ToppCluster and then displayed using Cytoscape.

We found that enriched KEGG pathways were most frequently associated with signal transduction (Apelin signaling pathway, mTOR signaling pathway, Hippo signaling pathway, WNT signaling pathway), cell growth and death (cell cycle, p53 signaling pathway, ferroptosis) tumorigenesis (viral carcinogenesis, proteoglycan in cancer, colorectal cancer, carbon metabolism, transcriptional mis-regulation in cancer, MicroRNAs in cancer), metabolism (beta-oxidation, acyl-CoA synthesis, fatty acid biosynthesis, pentose phosphate pathway, PRPP biosynthesis, ribose 5P => PRPP pathway) and cellular process (signaling pathway regulating pluripotency of stem cells) (Table S2: The KEGG pathways of downregulated miRNA gene targets in colorectal carcinoma). These enriched KEGG pathways displayed by Cytoscape point out that different downregulated miRNAs are involved in the same pathway, whereas a single downregulated miRNA targets genes which are involved in different pathways (Figure 3, Table S2: The KEGG pathways of downregulated miRNA gene targets in colorectal carcinoma). 

### 2.3. Association Analysis of Clinic-Pathological Features and miRNAs Expression Level 

Clinic-pathological and molecular data of CRC patients are summarized in Table 2. The median (interquartile range, IQR) time of survival was 31.5 (27.5–38.5) months. The anatomical site of the lesion was in the right colon in the majority of the cases (24, 54.6%). More than half of the patients showed advanced stage of the disease (stage III and stage IV: 46.5% and 14%, respectively) and a histologic grade 2 (30, 68.2%). *KRAS* mutation was found in 38.6% of the cases. The following miRNAs were down-regulated in the majority of the patients: miR-133a, miR-139-5p, miR-133b, miR-149-5p, miR-489, miR-145-5p, miR-150-5p; only miR-224-5p was upregulated. 

We summarize the association between CRC patients with variation of expression levels (defined by the fold change (FC) of each tumor) of miR-224-5p and miR-489 and clinic-pathological features of tumors in Table 3. MiR-224-5p overexpression was significantly associated with weak lymph node involvement, conversely it increases the presence of metastasis (85.7%, *p* value: 0.004 and 91.9%, *p* value: 0.006, respectively). Low miR-489 expression level was prevalently associated with patients aged >66 years (78.4%; *p* value: 0.002). No significant association between these miRNA expression levels and the other clinic-pathological features analyzed were found (Table 3).

The logistic regression analysis aimed at evaluating the association between miRNA expression and the selected covariates found a significant relationship between miR-224-5p and tumor stage IV (odds ratio (OR): 0.1; *p* value: 0.02) (Table 4). A similar analysis did not show statistically significant results for any other miRNA (Table 4).

**Figure 4:**
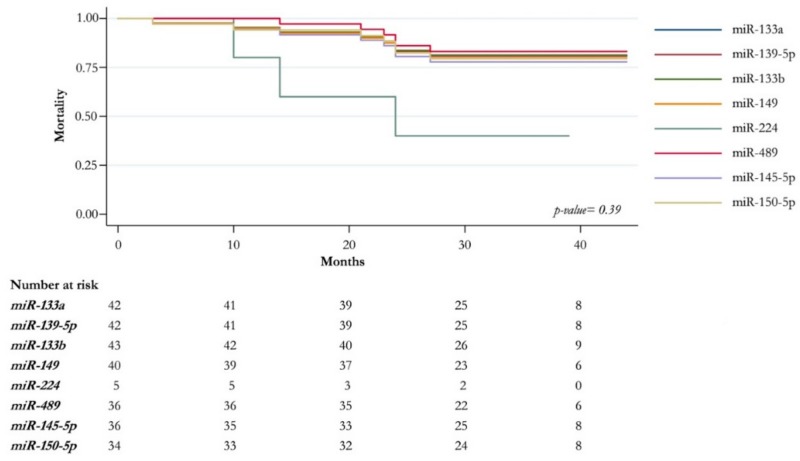
Survival analysis for miRNAs deregulated in CRC. Overall survival is shown according to up and down expression level (fold change) of miRNAs deregulated.

A Kaplan–Meier survival analysis to evaluate the clinical prognostic significance of deregulated miRNAs in CRC patients showed that the expression of miR-133a, miR133b, miR-145-5p, miR-139-5p, miR-149-5p, miR-489, and miR-150-5p was not related to the difference in patients’ survival (log-rank test; *p* value: 0.39). Moreover, the miR-224-5p upregulation seems to be associated with poor patients’ survival, although it does not show a significant *p* value (Figure 4).

## 3. Discussion

Tumorigenesis and CRC progression are characterized by a multistep processes and deregulation by oncogenes and oncosuppressor genes of multiple molecular networks. Based on the molecular and pathological characteristics of the tumor, they can be classified into five main groups [12]. It is essential to investigate the mechanisms of cellular regulatory networks and miRNA–mRNA interaction known to be involved in the pathogenesis of cancer. miRNAs show a simple mode of action and are able to regulate multiple genes involved in different pathways, the identification of which could improve diagnostic, prognostic and therapeutic approaches in CRC patients.

In the present study, an integrated signature of 19 downregulated and one overexpressed miRNAs was identified in CRC compared to paired NCT. Interestingly, some of the 20 miRNAs’ integrated signatures have been reported individually in literature as deregulated in CRC. Our data highlight their simultaneous deregulation in the same tumor. Moreover, we identified a strong positive or negative miRNA–miRNA correlation between downregulated miRNA and overexpressed miRNAs, respectively. Rarely a single miRNA is recognized as a specific biomarker. Recently, miR-576-5p and miR-20a have been identified as individual classifiers in CRC patients who may develop liver metastases, but the combination of the two, or better yet six, miRNAs has offered a far more effective model [13]. The mechanisms that control the progression of CRC may depend on the deregulation of multiple miRNAs resulting in complex dysfunction of cellular molecular networks. 

We identified gene targets for each deregulated miRNA by anti-correlation analysis between the 20 identified miRNAs and RNA-Seq data of 47 CRC and NCT [11]. An enrichment analysis of gene targets controlled by downregulated miRNAs in CRC have brought to light 25 genes known to relate to the pathogenesis of CRC. Our results further established their regulatory miRNAs and the multiple pathways involved (cell growth and death, and cell metabolism). 

Specifically, our results showed that eight downregulated miRNAs (let-7c, let-7e, miR-16-5p, miR-133b, miR-150-5p, miR-200c-3p, miR-222-3p, and miR-191-5p) operate epigenetic regulation of genes involved in the major cell growth and death pathways controlled by a complex network of regulators. This deregulation is in charge of the transformation of normal cells to neoplastic cells [14]. We underlined that the expression levels of different gene targets were modulated by more miRNAs (see Table S2: The KEGG pathways of downregulated miRNA gene targets in colorectal carcinoma).

Our data showed overexpression of the *CCND1* gene in CRC because of downregulation of let-7c, let-7e and miR-16-5p, which were identified as epigenetic modulators of *CCND1* mRNA. 

The CCND1 protein is a major cell cycle regulator of CDKs during the G1-S transition; consequently, the overexpression of *CCND1* implies the contemporary deregulation of different cell growth and death pathways. Different genetic aberrations of the *CCND1* gene have been related to tumorigenesis [15] and overexpression of this gene was observed in CRC, showing that increased levels of CCND1 proteins were significantly correlated with lymph nodes and distant metastases, and higher clinical stages [16,17]. Finally, Tong et al. demonstrated that the ectopic expression of miR-466 in SW-620 cells suppresses cell proliferation and migration/invasion, by decreasing the *CCND1* expression, which induces G0/G1 arrest [18]. 

We revealed *NKD1* overexpression (modulated by let-7c and let-7e) and *FZD3* (modulated by miR-222-3p and miR-200-3p), which are key determinants of the b-catenin-independent WNT pathways. This could impact on CRC tumorigenesis leading to a sustained increase in expression of alternative WNT-response pathways, such as WNT/planar cell polarity (PCP) and WNT/canonical, respectively. Over-expression of *NKD1* and *AXIN2* correlates with the activated WNT/β-catenin pathway in cell lines and in the human colon tumor [19]. It could be assumed that expression levels of *FZD3*, *NDK1* and *AXIN2* may be markers for the activation of the β-catenin WNTpathway in CRC. 

*HMGA2*, a target in our study of let-7c and let-7e, belongs to the non-histone chromosomal high mobility group protein family, which binds to AT-rich DNA, and stretches and modulates gene expression as architectural transcription factor [20]. Madison et al. have demonstrated that the *HMGA2* gene was the most highly induced target of let-7 in the context of intestinal homeostasis, and its protein expression levels were dramatically increased in intestinal tumors [21]. The overexpression of *HMGA2*, associated with the metastatic process, has a strongly negative impact on the survival of CRC patients [22]. Another target of let-7 is the *IGF2BP1* gene, highly expressed during the developmental process. Its overexpression and/or de novo synthesis are observed in various tumors [23]. Interestingly, Busch et al. have recently shown that *IGF2BP1* supports expression of itself, as well as of *LIN28B* and *HMGA2*, recruits these let-7 mRNA targets in mRNPs, and shields them from let-7 attack. In these conditions, *IGF2BP1*, *LIN28B* and *HMGA2* form a partially self-sustaining oncogenic triangle, where *HMGA2* enhances cell growth, and *IGF2BP1* and *LIN28B* promote the migratory and self-renewal potential of ovarian cancer cells [24]. We think that the downregulation of let-7c and let-7e might promote the self-sustaining oncogenic triangle, which supports the growth and progression of CRC. Moreover, the *HMGA2–LIN28B–IGF2BP1* triangle might be considered a promising strategy in cancer treatment. 

The *YAP1* gene was shown as a target of miR-16-5p and miR-200-3p, and encodes a protein acting as a transcriptional regulator of the Hippo pathway, whose overexpression or hyperactivation causes overgrowth in multiple tissues, including the liver, gastrointestinal tract, skin and heart [25,26,27]. Moreover, in cancer cell lines, *YAP* overexpression promotes resistance to apoptosis and anoikis induced by chemotherapeutic agents [28,29,30]. Extensive deregulation of miRNA signatures can lead to complex protein aberrations that could be responsible for the progression and aggressiveness of the tumors. Crosstalk mechanisms have shown that WNT and Hippo pathways intersect at two points: phosphorylated TAZs could activate DVL and thereby repress β-catenin, whereas YAPs could form a physical complex with β-catenin and cooperatively regulate transcription [31]. 

The *APLN* mRNA represents a miR-16-5p target which affects an epigenetic mechanism of gene deregulation. In tumor cells, *APLN* gene expression is induced by hypoxia [32], but its overexpression can be hypoxia-independent when caused by genomic alterations [33]. The secretion of APLN protein by tumor cells induces AKT phosphorylation and caspase inhibition influencing the growth of CRC [33]. Moreover, it could act in a paracrine manner on endothelial cells promoting tumor angiogenesis [32]. Therefore, *APLN* overexpression could represent an interesting pharmacological target by blocking inhibition of tumor growth by increasing apoptosis and decreasing tumor vascularization.

Analyzing CRC transcriptome profiling, we identified recurrent deregulation of the *MAD2L1* gene, caused by overexpression, missense mutations and alternative splicing. Here we demonstrated that *MAD2L1* overexpression depends on miR-133b downregulation. MAD2L1 protein is involved in stimulating and executing spindle checkpoint processes that lead to chromosomal instability. This important phenotype of the carcinogenesis plays a causative role in CRC initiation and progression [34]. According to the high frequency of Chromosomal Instability (CIN) in CRC, we think that *MAD2L1* could be used as a new potential prognostic biomarker. The key downstream effector of spindle assembly checkpoint might be particularly relevant as a putative therapeutic target.

Feng et al. described downregulation of miR-145-5p in CRC cell lines identifying *FSCN1* as a target of this miRNA [35]. Our results identified that the metastatic oncogenic *FSCN1* mRNA is a target of miR-145-5p and miR-133b: this combined downregulation could represent a potential prognostic biomarker for CRC.

In our study, intriguing results involve the *TP53* gene modulated by let-7c, let-7e and miR-150-5p. Recently, the negative regulator role of *TP53* in ferroptosis has been deeply investigated showing a pro-survival function of *TP53* by inhibition of ferroptosis cell death in human CRC cells and in vivo experiments through the regulation of DPP4 localization and activity [36]. The complex TP53-DPP4 increases *SLC7A11* expression level and contributes to ferroptosis resistance in *TP53* wild-type CRC cells [36]. The epigenetic regulation of *TP53* and *SLC7A11* expression levels identified here and the role of these genes in ferroptosis induction could have significant prognostic and therapeutic advantages for CRC. 

Cancer cells frequently share metabolic abnormalities, allowing them to accumulate metabolic intermediates as a source for cell growth and proliferation [37]. Our experience highlights a deregulation of glucose and lipid metabolism in CRC cells. Specifically, we identify that the downregulated let-7c, let-7e, miR-133a, miR-133b, miR-191-5p and miR-222-3p target *PRPS1-2* and *ACSL6* mRNA, overexpressed in CRC.

*PRPS1* and *PRPS2* genes are key enzymes in the synthesis of purine, pyrimidine and various coenzymes, regulating the pentose phosphate pathway (PPP). Our results suggest that their overexpression in neoplastic cells influences DNA synthesis and cell growth. We assume that these cellular functions might be strengthened by overexpression of the *RRM2* gene, which is a target of let-7c and let-7e. This small subunit of the RR complex, well known for its role in DNA synthesis, provides a balanced supply of precursors for DNA synthesis and repair. *RRM2* is also involved in the regulation of tumor angiogenesis through downregulation of the anti-angiogenic TSP-1 [38].

Cancer cells frequently show altered lipid metabolism with an increase of fatty acids (FA) de novo synthesis offering advantages in proliferation, progression and metastasis [37,39]. Specifically, the ACSL protein family generates the cellular bioactive FA-CoA pools. The overexpression of *ACSL6* in our tumor cohort agrees with an *ACSL6* downregulation in most forms of cancers, except CRC [40]. *ACSL6*, *PRPS1* and *PRPS2* genes represent potential therapeutic targets to limit cancer cell growth using specific inhibitors, which could re-program cancer cell metabolism by reducing FA. Specific inhibitors for different isoforms are already available for ACSL, such as triacsin C and thiazolidinediones, specifically for ACSL1 and ACSL4 [41,42].

Finally, we emphasize that the overexpression of miR-224 favors the progression of CRCs by promoting distant metastases but not lymph node metastases. This prognostic factor was significantly associated with higher tumor stages, and correlated with poor survival, although it did not achieve statistical significance. 

## 4. Materials and Methods 

### 4.1. Patients and Samples

We screened more than 100 anonymized and consecutive patients diagnosed with CRC that underwent surgical resection at the Surgery Unit of University of Sassari from June 2014 to December 2015. Forty-seven primary colorectal carcinoma and related NCT were enrolled in the present study. We excluded patients who received neoadjuvant chemo and/or radiotherapy and showed multiple recurrence and/or CRC familiarity. Tissue samples were collected in RNAlater solution and stored at −80 °C. All tumors were critically reviewed and assessed by a pathologist, according to WHO criteria [43]. All clinic-pathological and follow-up data were obtained from medical records. The follow-up started at the time of diagnosis (June 2014–December 2015) and ended on 28 February 2018. Written informed consent was obtained from each patient and the protocol was reviewed and approved by the Azienda Sanitaria Locale Sassari Bioethics Committee (n. 2032/CE, 13/05/2014). The study was conducted in accordance with the code of ethics of the World Medical Association (Declaration of Helsinki). 

### 4.2. Human miRNA Card Array and Quantitative Real-Time PCR

Total RNA was extracted by neoplastic and non-neoplastic tissues, by homogenizing 100 mg of tissue in 1 mL of QIAzol lysis reagent (QIAGEN, Hilden, Germany) and subsequently by means of miRNeasy Mini Kit (QIAGEN) in accordance with the manufacturer’s instructions, as previously described [44]. The high-throughput miRNA expression profiling was first performed on 8 pairs of CRC and paired NCT from patients with CRC, and these samples became part of the validation cohort. We have used the TaqMan^®^ Array Human MicroRNA Card A set v3.0 (Thermo Fisher Scientific, Waltham, MA, USA), a high throughput PCR-based miRNA array, which enables analysis of 377 miRNA assays present in the miRBase version 18.0. Moreover, the card A contains 3 endogenous controls (MammU6, RUN44, and RUN48) for relative quantization, of which MammU6 was present in 4 replicates while the other 2 controls appeared just once, and an assay unrelated to any mammalian species, ath-miR-159a, as a negative control. The differential expression of significantly deregulated miRNAs (*p* value < 0.05) was further validated by RT-qPCR in all datasets (47 CRC and 47 NCT) following the procedures described in Uva et al. [44]. MiRNA U6 was used as reference for normalizing miRNA expression. All reactions were performed in triplicate.

### 4.3. Experimental Identification of miRNA Gene Targets, Gene Ontology and Pathways Mapping 

The gene targets of CRC related to differentially expressed miRNAs were predicted by 7 algorithms: DianaMicroT_strict [45], miRanda-mirSVR_S_C [46], MirTarget2 [47], picTar_chicken [48], PITA_Top [49], starBase [50] and TargetScan_v6.2 [51]. Experimentally validated targets were identified by literature and/or from miRecords [52] and mirTarBase v4.5 [53] databases. Comparisons of gene targets lists were performed with custom scripts using the computing environment R [54]. Targets predicted by at least 1 of the 7 algorithms or experimentally validated (i.e., reported in at least 1 database or in literature) were selected for subsequent analysis.

Predicted miRNA targets were then compared with the transcriptional profiles of CRC and NCT samples obtained by Next Generation Sequencing experiments [11], searching for significant overlaps between predicted targets of miRNA and mRNA whose expression was the opposite of that of miRNAs. The mRNAs with inverse expression were defined as anti-expressed. A hypergeometric test was applied for statistical evaluation of the significance of the overlap.

To inspect the function of the differentially expressed miRNAs, the gene targets were submitted to Gene Ontology and KEGG pathway enrichment analysis using ToppCluster [55]. Terms with false discovery rate (FDR)-corrected enrichment *p* values <0.05 were considered. ToppCluster results were displayed using Cytoscape [56].

### 4.4. Statistical Analysis

Relative miRNA expression was calculated using the comparative cycle threshold (2^−ΔΔCt^) method [57]. Ct values were normalized using the quantile normalization method. An unsupervised hierarchical clustering, using Pearson’s correlation as distance measure and average linkage as agglomerative algorithm, was used to assess which samples clustered together based on their expression profiles. miRNAs with statistically significant changes in expression were identified by SAM analysis [58]. Differences with FDR-corrected *p* value <0.05 were retained as statistically significant. The analyses were performed in R using the samr package for differential expression analysis [54]. To estimate the relationships between miRNA expression levels the Pearson’s correlation coefficient was calculated by R software. 

An ad hoc electronic form (Excel, Microsoft) was used to collect demographic, epidemiologic, molecular, and clinical variables. Qualitative and quantitative variables were summarized with absolute and relative (percentage) frequencies and medians following their non-parametric distribution. The selected variables were evaluated in those patients with down- and upregulation of miR-224-5p, as well as in those with down- and upregulation of miR-489. Qualitative variables were evaluated with the chi-squared or Fisher exact test, when appropriate; quantitative variables were compared with the Mann–Whitney test. Logistic regression analyses were carried out to assess the relationship between the above-mentioned variables and the upregulation of miR-224-5p and miR-489. Survival analysis was performed for assessing the time-to-death of CRC patients with a down-regulation of all miRNA in CRC compared to NCT. Survival curves were estimated by the Kaplan–Meier method and the difference between the curves was evaluated by Log-Rank test (Mantel–Cox). The analyses were performed using STATA v15 (STATA Corp., College Station, TX, USA). For all statistical tests, *p* values <0.05 were considered significant. 

## 5. Conclusions

The present study identified an integrated signature of 20 deregulated miRNAs in CRC patients, showing a strong positive or negative miRNA–miRNA correlation based on expression levels. We believe that the biology and progression of CRC could depend on deregulation of multiple miRNAs which determine a complex dysfunction of cellular molecular networks. We experimentally identified gene targets for each deregulated miRNA, bringing to light 25 genes operating as members of pathways known to lead to cell growth and death, as well as cell metabolism. Although these genes are known to relate to the pathogenesis of CRC, our results further established their epigenetic mechanism of control and defined the multiple pathways involved. These miRNA integrated signatures and gene targets could be evaluated as potential prognostic biomarkers and/or therapeutic targets.

## Figures and Tables

**Table 1:** Correlation between miRNAs expression levels.

	miR-139-5p	miR-133b	miR-149	miR-224	miR-489	miR-145	miR-150
miR-133a	0.87 **	0.90 **	0.77 **	−0.52 **	0.44 *	0.74 **	0.67 **
miR-139-5p		0.81 **	0.85 **	−0.44 *	0.44 *	0.64 **	0.69 **
miR-133b			0.77 **	−0.34 *	0.37 *	0.82 **	0.71 **
miR-149				−0.22	0.38 *	0.57 **	0.49 *
miR-224					−0.19	−0.27	−0.31
miR-489						0.14	0.31
miR-145							0.64 **

** *p* value <0.001; * *p* value <0.05.

**Table 2:** Descriptive analysis of qualitative and quantitative variables.

Alive at 28/02/2018, *n* (%)		35 (79.6)
Median (IQR) time of survival, months		31.5 (27.5–38.5)
Localization, *n* (%)	Right	24 (54.6)
	Left	14 (31.8)
	Rectum	6 (13.6)
Tumor stage, *n* (%)	I	11 (25.6)
	II	6 (14.0)
	III	20 (46.5)
	IV	6 (14.0)
Histologic grade, *n* (%)	G1	2 (4.6)
	G2	30 (68.2)
	G3	12 (27.3)
Tumor infiltrating lymphocytes, *n* (%)		13 (31.0)
*KRAS* mutational status, *n* (%)		17 (38.6)
MiR-133a, *n* (%)	Down	43 (97.7)
	Up	1 (2.3)
MiR-139-5p, *n* (%)	Down	43 (97.7)
	Up	1 (2.3)
MiR-133b, *n* (%)	Down	44 (100.0)
	Up	0 (0.0)
MiR-149-5p, *n* (%)	Down	41 (97.6)
	Up	1 (2.4)
MiR-224-5p, *n* (%)	Down	6 (14.0)
	Up	37 (86.1)
MiR-489, *n* (%)	Down	37 (86.1)
	Up	6 (14.0)
MiR-145-5p, *n* (%)	Down	37 (100.0)
	Up	0 (0.0)
MiR-150-5p, *n* (%)	Down	35 (94.6)
	Up	2 (5.4)

*n* = number; IQR = interquartile ranges.

**Table 3:** Univariate analysis to assess the relationship between miR-224-5p and miR-489 expression level deregulation, and clinic-pathological and molecular features.

Variables		miR-224-5p		miR-489	
		OR (95% CI)	*p* Value	OR (95% CI)	*p* Value
Localization	Right	1.2 (0.2–6.6)	0.85	-	-
	Left	0.4 (0.1–2.4)	0.34	0.4 (0.1–2.3)	0.30
	Rectum	-	-	5.4 (0.9–34.2)	0.07
Tumor stage	I	-	-	0.5 (0.1–5.0)	0.57
	II	0.8 (0.1–8.4)	0.86	-	-
	III	1.8 (0.3–11.0)	0.53	1.3 (0.2–7.1)	0.80
	IV	0.1 (0.0–0.7)	**0.02**	4 (0.6–29.3)	0.17
Histologic grade	G1	-	-	7.2 (0.4–134.2)	0.19
	G2	1.2 (0.2–7.4)	0.86	0.9 (0.1–5.3)	0.86
	G3	0.6 (0.1–4.1)	0.64	0.5 (0.1–5.2)	0.59
Tumor infiltrating lymphocytes		0.7 (0.1–4.5)	0.67	-	-
*KRAS* mutational status		3.4 (0.4–32.2)	0.28	4.2 (0.7–26.0)	0.13

OR: odds ratio; CI: confidence interval. The *p* values are bold where they are less than or equal to the significance level of 0.05.

**Table 4:** Clinic-pathological and molecular data of CRC patients according to miR-224-5p and miR-489 expression level deregulation.

Variables	miR-224-5p			miR-489		
	Down	Up	*p* Value	Down	Up	*p* Value
**Gender**						
Male, *n* (%)	4 (66.7)	23 (62.2)	0.83	24 (64.9)	3 (50.0)	0.48
Female, *n* (%)	2 (33.3)	14 (37.8)	0.83	13 (35.1)	3 (50.0)	0.48
**Age at diagnosis**						
Under 65 years, *n* (%)	2 (33.3)	11 (29.7)	0.86	8 (21.6)	5 (83.3)	**0.002**
Over 66 years, *n* (%)	4 (66.7)	26 (70.3)	0.86	29 (78.4)	1 (16.7)	**0.002**
**Localization**						
Right, *n* (%)	3 (50.0)	20 (54.1)	1.0	21 (56.7)	2 (33.3)	0.39
Left, *n* (%)	3 (50.0)	11 (29.7)	0.37	10 (27.0)	4 (66.7)	0.08
Rectum, *n* (%)	0 (0.0)	6 (16.2)	0.57	6 (16.2)	0 (0.0)	0.57
**Histologic grade**						
G1–G2, *n* (%)	4 (66.7)	28 (75.7)	0.64	27 (73.0)	5 (83.3)	0.59
G3, *n* (%)	2 (33.3)	9 (24.3)	0.64	10 (27.0)	1 (16.7)	0.59
**Depth of invasion**						
T1–T2, *n* (%)	0 (0.0)	14(37.8)	0.07	12 (32.4)	2 (33.3)	0.97
T3–T4, *n* (%)	6 (100.0)	23 (62.2)	0.07	25 (67.6)	4 (66.7)	0.97
**Nodal status**						
N0–N1, *n* (%)	2 (33.3)	30 (85.7)	**0.004**	29 (80.6)	3 (60.0)	0.30
N2–N3, *n* (%)	4 (66.7)	5 (14.3)	**0.004**	7 (19.4)	2 (40.0)	0.30
**Distant metastasis**						
Present, *n* (%)	3 (50.0)	34 (91.9)	**0.006**	33 (89.2)	4 (66.7)	0.14
Absent, *n* (%)	3 (50.0)	3 (8.1)	**0.006**	4 (10.8)	2 (33.3)	0.14
**Tumor stage**						
I–II, *n* (%)	1 (16.7)	16 (44.4)	0.20	16 (44.4)	1 (16.7)	0.20
III–IV, *n* (%)	5 (83.3)	20 (55.6)	0.20	20 (55.6)	5 (83.3)	0.20
**Tumor infiltrating lymphocytes**						
Present, *n* (%)	2 (40.0)	11 (30.6)	0.67	13 (36.1)	0 (0.0)	0.10
Absent, *n* (%)	3 (60.0)	25 (69.4)	0.67	23 (63.9)	5 (100.0)	0.10
**Neoplastic embolization**						
Present, *n* (%)	3 (60.0)	9 (25.0)	0.11	10 (27.8)	2 (40.0)	0.57
Absent, *n* (%)	2 (40.0)	27 (75.0)	0.11	26 (72.2)	3 (60.0)	0.57
**Perineural invasion**						
Present, *n* (%)	1 (20.0)	2 (5.6)	0.25	3 (8.3)	0 (0.0)	0.50
Absent, *n* (%)	4 (80.0)	34 (94.4)	0.25	33 (91.7)	5 (100.0)	0.50
**KRAS mutational status**						
Wild type, *n* (%)	5 (83.3)	22 (59.5)	0.26	25 (67.6)	2 (33.3)	0.11
Mutation, *n* (%)	1 (16.7)	15 (40.5)	0.26	12 (32.4)	4 (66.7)	0.11

The *p* values are bold where they are less than or equal to the significance level of 0.05.

## Supplementary Materials

Supplementary materials can be found at https://www.mdpi.com/1422-0067/20/16/4067/s1.

## Abbreviations

CRC	Colorectal cancer
NCT	Normal colon tissue
MiRNAs	MicroRNAs
TLDA	TaqMan^®^ Array Human MicroRNA technology
SAM	Statistical Analysis of Microarray
DE	Differentially expressed
GO	Gene Ontology
IQR	Interquartile range
FC	Fold Change
OR	Odds Ratio
CI	Confidence Interval
PCP	Planar cell polarity
PPP	Pentose phosphate pathway
FA	Fatty acids
CIN FDR	Chromosomal Instability False Discovery Rate

## References

[1] Siegel R.L., Miller K.D., Fedewa S.A., Ahnen D.J., Meester R.G.S., Barzi A., Jemal A. (2017). Colorectal cancer statistics, 2017. CA. Cancer J. Clin..

[2] Brenner H., Kloor M., Pox C.P. (2014). Colorectal Cancer. Lancet..

[3] Riihimaki M., Hemminki A., Sundquist J., Hemminki K. (2016). Patterns of metastasis in colon and rectal cancer. Sci. Rep..

[4] Guo L., Lu Z. (2010). The fate of miRNA* strand through evolutionary analysis: Implication for degradation as merely carrier strand or potential regulatory molecule?. PLoS ONE.

[5] He L., Hannon G.J. (2004). MicroRNAs: Small RNAs with a big role in gene regulation. Nat. Rev. Genet..

[6] Peng Y., Croce C.M. (2016). The role of microRNAs in human cancer. Signal Transduct. Target. Ther..

[7] Bao Y., Chen Z., Guo Y., Feng Y., Li Z., Han W., Wang J., Zhao W., Jiao Y., Li K. (2014). Tumor suppressor MicroRNA-27a in colorectal carcinogenesis and progression by targeting SGPP1 and Smad2. PLoS ONE.

[8] Liu L., Chen L., Xu Y., Li R., Du X. (2010). MicroRNA-195 promotes apoptosis and suppresses tumorigenicity of human colorectal cancer cells. Biochem. Biophys. Res. Commun..

[9] Hollis M., Nair K., Vyas A., Chaturvedi L.S., Gambhir S., Vyas D. (2015). MicroRNAs potential utility in colon cancer: Early detection, prognosis, and chemosensitivity. World J. Gastroenterol..

[10] Yang J., Ma D., Fesler A., Zhai H., Leamniramit A., Li W., Wu S., Ju J. (2017). Expression analysis of microRNA as prognostic biomarkers in colorectal cancer. Oncotarget.

[11] Pira G., Uva P., Scanu A., Cossu Rocca P., Uleri E., Piu C., Porcu A., Carru C., Manca A., Persico I. (2019). Landscape of transcriptome variations uncovering known and novel driver events in colorectal carcinoma. Sci. Rep..

[12] Arends M.J. (2013). Pathways of colorectal carcinogenesis. Appl. Immunohistochem. Mol. Morphol..

[13] Makondi P.T., Wei P.L., Huang C.Y., Chang Y.J. (2019). Development of novel predictive miRNA/ target gene pathways for colorectal cancer distance metastasis to the liver using a bioinformatic approach. PLoS ONE.

[14] Slattery M.L., Herrick J.S., Mullany L.E., Samowitz W.S., Sevens J.R., Sakoda L., Wolff R.K. (2017). The co-regulatory networks of tumor suppressor genes, oncogenes, and miRNAs in colorectal cancer. Genes Chromosom. Cancer.

[15] Baldin V., Lukas J., Marcote M.J., Pagano M., Draetta G. (1993). Cyclin D1 is a nuclear protein required for cell cycle progression in G1. Genes Dev..

[16] Al-Kuraya K., Novotny H., Bavi P., Siraj A.K., Uddin S., Ezzat A., Al Sanea N., Al-Dayel F., Al-Mana H., Sheikh S.S. (2007). HER2, TOP2A, CCND1, EGFR and C-MYC oncogene amplification in colorectal cancer. J. Clin. Pathol..

[17] Balcerczak E., Pasz-Walczak G., Kumor P., Panczyk M., Kordek R., Wierzbicki R., Mirowski M. (2005). Cyclin D1 protein and CCND1 gene expression in colorectal cancer. Eur. J. Surg. Oncol..

[18] Tong F., Ying Y., Pan H., Zhao W., Li H., Zhan X. (2018). MicroRNA-466 (miR-466) functions as a tumor suppressor and prognostic factor in colorectal cancer (CRC). Bosn. J. Basic Med. Sci..

[19] Yan D., Wiesmann M., Rohan M., Chan V., Jefferson A.B., Guo L., Sakamoto D., Caothien R.H., Fuller J.H., Reinhard C. (2001). Elevated expression of axin2 and hnkd mRNA provides evidence that Wnt/ beta -catenin signaling is activated in human colon tumors. Proc. Natl. Acad. Sci. USA.

[20] Reeves R. (2001). Molecular biology of HMGA proteins: Hubs of nuclear function. Gene.

[21] Madison B.B., Jeganathan A.N., Mizuno R., Winslow M.M., Castells A., Cuatrecasas M., Rustgi A.K. (2015). Let-7 Represses Carcinogenesis and a Stem Cell Phenotype in the Intestine via Regulation of Hmga2. PLoS Genet..

[22] Wang X., Liu X., Li A.Y.J., Chen L., Lai L., Lin H.H., Hu S., Yao L., Peng J., Loera S. (2011). Overexpression of HMGA2 promotes metastasis and impacts survival of colorectal cancers. Clin. Cancer Res..

[23] Lederer M., Bley N., Schleifer C., Hüttelmaier S. (2014). The role of the oncofetal IGF2 mRNA-binding protein 3 (IGF2BP3) in cancer. Semin. Cancer Biol..

[24] Busch B., Bley N., Müller S., Glaß M., Misiak D., Lederer M., Vetter M., Strauß H.G., Thomssen C., Hüttelmaier S. (2016). The oncogenic triangle of HMGA2, LIN28B and IGF2BP1 antagonizes tumor-suppressive actions of the let-7 family. Nucleic Acids Res..

[25] Camargo F.D., Gokhale S., Johnnidis J.B., Fu D., Bell G.W., Jaenisch R., Brummelkamp T.R. (2007). YAP1 Increases Organ Size and Expands Undifferentiated Progenitor Cells. Curr. Biol..

[26] Schlegelmilch K., Mohseni M., Kirak O., Pruszak J., Rodriguez J.R., Zhou D., Kreger B.T., Vasioukhin V., Avruch J., Brummelkamp T.R. (2011). Yap1 acts downstream of α-catenin to control epidermal proliferation. Cell.

[27] Heallen T., Zhang M., Wang J., Bonilla-Claudio M., Klysik E., Johnson R.L., Martin J.F. (2011). Hippo pathway inhibits Wnt signaling to restrain cardiomyocyte proliferation and heart size. Science.

[28] Zhao B., Li L., Wang L., Wang C.Y., Yu J., Guan K.L. (2012). Cell detachment activates the Hippo pathway via cytoskeleton reorganization to induce anoikis. Genes Dev..

[29] Tapon N., Harvey K.F., Bell D.W., Wahrer D.C.R., Schiripo T.A., Haber D.A., Hariharan I.K. (2002). Salvador promotes both cell cycle exit and apoptosis in Drosophila and is mutated in human cancer cell lines. Cell.

[30] Zhang X., George J., Deb S., Degoutin J.L., Takano E.A., Fox S.B., Bowtell D.D.L., Harvey K.F. (2011). The Hippo pathway transcriptional co-activator, YAP, is an ovarian cancer oncogene. Oncogene.

[31] Varelas X., Miller B.W., Sopko R., Song S., Gregorieff A., Fellouse F.A., Sakuma R., Pawson T., Hunziker W., McNeill H. (2010). The Hippo Pathway Regulates Wnt/β-Catenin Signaling. Dev. Cell.

[32] Sorli S.C., Le Gonidec S., Knibiehler B., Audigier Y. (2007). Apelin is a potent activator of tumour neoangiogenesis. Oncogene.

[33] Picault F.X., Chaves-Almagro C., Projetti F., Prats H., Masri B., Audigier Y. (2014). Tumour co-expression of apelin and its receptor is the basis of an autocrine loop involved in the growth of colon adenocarcinomas. Eur. J. Cancer.

[34] Cahill D.P., Lengauer C., Yu J., Riggins G.J., Willson J.K., Markowitz S.D., Kinzler K.W., Vogelstein B. (1998). Mutations of mitotic checkpoint genes in human cancers. Nature.

[35] Feng Y., Zhu J., Ou C., Deng Z., Chen M., Huang W., Li L. (2014). MicroRNA-145 inhibits tumour growth and metastasis in colorectal cancer by targeting fascin-1. Br. J. Cancer.

[36] Xie Y., Zhu S., Song X., Sun X., Fan Y., Liu J., Zhong M., Yuan H., Zhang L., Billiar T.R. (2017). The Tumor Suppressor p53 Limits Ferroptosis by Blocking DPP4 Activity. Cell Rep..

[37] Currie E., Schulze A., Zechner R., Walther T.C., Farese R.V. (2013). Cellular Fatty Acid Metabolism and Cancer. Cell Metab..

[38] Zhang K., Hu S., Wu J., Chen L., Lu J., Wang X., Liu X., Zhou B., Yen Y. (2009). Overexpression of RRM2 decreases thrombspondin-1 and increases VEGF production in human cancer cells in vitro and in vivo: Implication of RRM2 in angiogenesis. Mol. Cancer.

[39] Santos C.R., Schulze A. (2012). Lipid metabolism in cancer. FEBS J..

[40] Chen W.C., Wang C.Y., Hung Y.H., Weng T.Y., Yen M.C., Lai M.D. (2016). Systematic analysis of gene expression alterations and clinical outcomes for long-chain acyl-coenzyme A synthetase family in cancer. PLoS ONE.

[41] Van Horn C.G., Caviglia J.M., Li L.O., Wang S., Granger D.A., Coleman R.A. (2005). Characterization of recombinant long-chain rat Acyl-CoA synthetase isoforms 3 and 6: Identification of a novel variant of isoform 6. Biochemistry.

[42] Mashima T., Oh-hara T., Sato S., Mochizuki M., Sugimoto Y., Yamazaki K., Hamada J.I., Tada M., Moriuchi T., Ishikawa Y. (2005). p53-defective tumors with a functional apoptosome-mediated pathway: A new therapeutic target. J. Natl. Cancer Inst..

[43] Hamilton S.R., Bosman F.T., Boffetta P., Boman F.T., Carneiro F., Hruban R.H., Theise N.D. (2010). Carcinoma of the colon and rectum. WHO Classification of Tumours of the Digestive System.

[44] Uva P., Cossu-Rocca P., Loi F., Pira G., Murgia L., Orrù S., Floris M., Muroni M.R., Sanges F., Carru C. (2018). miRNA-135b contributes to triple negative breast cancer molecular heterogeneity: Different expression profile in Basal-like versus non-Basal-like phenotypes. Int. J. Med. Sci..

[45] Paraskevopoulou M.D., Georgakilas G., Kostoulas N., Vlachos I.S., Vergoulis T., Reczko M., Filippidis C., Dalamagas T., Hatzigeorgiou A.G. (2013). DIANA-microT web server v5.0: Service integration into miRNA functional analysis workflows. Nucleic Acids Res..

[46] Betel D., Wilson M., Gabow A., Marks D.S., Sander C. (2008). The microRNA.org resource: Targets and expression. Nucleic Acids Res..

[47] Wong N., Wang X. (2015). miRDB: An online resource for microRNA target prediction and functional annotations. Nucleic Acids Res..

[48] Krek A., Grün D., Poy M.N., Wolf R., Rosenberg L., Epstein E.J., MacMenamin P., Da Piedade I., Gunsalus K.C., Stoffel M. (2005). Combinatorial microRNA target predictions. Nat. Genet..

[49] Kertesz M., Iovino N., Unnerstall U., Gaul U., Segal E. (2007). The role of site accessibility in microRNA target recognition. Nat. Genet..

[50] Li J.H., Liu S., Zhou H., Qu L.H., Yang J.H. (2014). StarBase v2.0: Decoding miRNA-ceRNA, miRNA-ncRNA and protein-RNA interaction networks from large-scale CLIP-Seq data. Nucleic Acids Res..

[51] Lewis B.P., Burge C.B., Bartel D.P. (2005). Conserved seed pairing, often flanked by adenosines, indicates that thousands of human genes are microRNA targets. Cell.

[52] Xiao F., Zuo Z., Cai G., Kang S., Gao X., Li T. (2009). miRecords: An integrated resource for microRNA-target interactions. Nucleic Acids Res..

[53] Hsu S.D., Lin F.M., Wu W.Y., Liang C., Huang W.C., Chan W.L., Tsai W.T., Chen G.Z., Lee C.J., Chiu C.M. (2011). MiRTarBase: A database curates experimentally validated microRNA-target interactions. Nucleic Acids Res..

[54] Ihaka R., Gentleman R. (1996). R: A Language for Data Analysis and Graphics. J. Comput. Graph. Stat..

[55] Kaimal V., Bardes E.E., Tabar S.C., Jegga A.G., Aronow B.J. (2010). ToppCluster: A multiple gene list feature analyzer for comparative enrichment clustering and networkbased dissection of biological systems. Nucleic Acids Res..

[56] Shannon P., Markiel A., Ozier O., Baliga N.S., Wang J.T., Ramage D., Amin N., Schwikowski B., Ideker T. (2003). Cytoscape: A software environment for integrated models of biomolecular interaction networks. Genome Res..

[57] Livak K.J., Schmittgen T.D. (2001). Analysis of relative gene expression data using real-time quantitative PCR and the 2^−ΔΔCT^ method. Methods.

[58] Tusher V.G., Tibshirani R., Chu G. (2001). Significance analysis of microarrays applied to the ionizing radiation response. Proc. Natl. Acad. Sci. USA.

